# Adherence to HIV antiretroviral therapy among pregnant and postpartum women during the Option B+ era: 12‐month cohort study in urban South Africa and rural Uganda

**DOI:** 10.1002/jia2.25586

**Published:** 2020-08-20

**Authors:** Lynn T Matthews, Catherine Orrell, Mwebesa Bosco Bwana, Alexander C Tsai, Christina Psaros, Stephen Asiimwe, Gideon Amanyire, Nicholas Musinguzi, Kathleen Bell, David R Bangsberg, Jessica E Haberer, the META Study Investigators, Nomakhaya April, Alienah Mpahleni, Vivie Situlo, Speech Mzamo, Nomsa Ngwenya, Khosi Tshangela Regina Panda, Teboho Linda, Christine Atwiine, Sheila Moonight, Edna Tindimwebwa, Nicholas Mugisha, Peace Atwogeire, Vian Namana, Catherine Kyampaire, Gabriel Nuwagaba, Annet Kembabazi, Stephen Mugisha, Victoria Nanfuka, Anna Cross, Nicky Kelly, Daphne Moralie, Dolphina Cogill, Justus Ashaba, Zoleka Xapa, Mathias Orimwesiga, Elly Tuhanamagyezi, Don Bosco Mpanga, Leonia Kyarisima, Simone Kigozi, Edgar October, Silver Mugisha, Ibrahim Kiviiri, Norma Ware, Tumwesigye Elioda, Mark J Siedner, Ingrid T Katz, Monique Wyatt

**Affiliations:** ^1^ Department of Medicine University of Alabama at Birmingham Birmingham AL USA; ^2^ Department of Medicine Massachusetts General Hospital Boston MA USA; ^3^ Desmond Tutu Foundation Rondebosch South Africa; ^4^ Mbarara University of Science and Technology Mbarara Uganda; ^5^ Department of Psychiatry Massachusetts General Hospital Boston MA USA; ^6^ Center for Global Health Massachusetts General Hospital Boston MA USA; ^7^ Harvard Center for Population and Development Studies Boston MA USA; ^8^ Kabwohe Clinical Research Center (KCRC) Kabwohe Uganda; ^9^ Makerere‐Mbarara Universities Joint AIDS Program (MJAP) Mbarara Uganda; ^10^ School of Public Health Oregon Health and Science University/Portland State University Portland OR USA

**Keywords:** adherence, ARV, Cohort studies, gender, Africa < Region, women, HIV, adherence, ARV, cohort studies, gender, Africa < Region, women, HIV

## Abstract

**Introduction:**

We conducted a cohort study to understand patterns of anti‐retroviral therapy (ART) adherence during pregnancy, postpartum and non‐pregnancy follow‐up among women initiating ART in public clinics offering Option B+ in rural Uganda and urban South Africa.

**Methods:**

We collected survey data, continuously monitored ART adherence (Wisepill), HIV‐RNA and pregnancy tests at zero, six and twelve months from women initiating ART in Uganda and South Africa, 2015 to 2017. The primary predictor of interest was follow‐up time categorized as pregnant (pregnancy diagnosis to pregnancy end), postpartum (pregnancy end to study exit) or non‐pregnancy‐related (neither pregnant nor postpartum). Fractional regression models included demographics and socio‐behavioural factors informed by the Behavioral Model for Vulnerable Populations. We evaluated HIV‐RNA at 12 months by ever‐ versus never‐pregnant status.

**Results:**

In Uganda, 247 women contributed 676, 900 and 1274 months of pregnancy, postpartum and non‐pregnancy‐related follow‐up. Median ART adherence was consistently ≥90%: pregnancy, 94% (interquartile range [IQR] 78,98); postpartum, 90% (IQR 70,97) and non‐pregnancy, 90% (IQR 80,98). Poorer adherence was associated with younger age (0.98% [95% CI 0.33%, 1.62%] average increase per year of age) and higher CD4 cell count (1.01% [0.08%, 1.94%] average decrease per 50 cells/mm^3^). HIV‐RNA was suppressed among 91% (N = 135) ever‐pregnant and 86% (N = 85) never‐pregnant women. In South Africa, 190 women contributed 259, 624 and 1247 months of pregnancy, postpartum and non‐pregnancy‐related follow‐up. Median adherence was low during pregnancy, 74% (IQR 31,96); postpartum, 40% (IQR 4,65) and non‐pregnancy, 77% (IQR 47,92). Poorer adherence was associated with postpartum status (22.3% [95%CI 8.6%, 35.4%] average decrease compared to non‐pregnancy‐related follow‐up) and less emotional support (1.4% [0.22%, 2.58%] average increase per unit increase). HIV‐RNA was suppressed among 57% (N = 47) ever‐pregnant and 86% (N = 93) never‐pregnant women.

**Conclusions:**

Women in rural Uganda maintained high adherence with 91% of ever‐pregnant and 86% of never‐pregnant women suppressing HIV‐RNA at 12 months. Women in urban South Africa struggled with adherence, particularly during postpartum follow‐up with median adherence of 40% and 57% of women with HIV‐RNA suppression at one year, suggesting a crisis for postpartum women with HIV in South Africa. Findings suggest that effective interventions should promote emotional support.

## 1 INTRODUCTION

In 2013, the WHO updated guidelines for prevention of mother to child transmission of HIV (PMTCT) to recommend antiretroviral treatment (ART) for pregnant or breastfeeding women living with HIV (WLWH), either during pregnancy and breast‐feeding periods only (“Option B”), or for life (“Option B+”), regardless of CD4 count. The goal was to simplify service delivery while reducing perinatal transmission in current and subsequent pregnancies, drug resistance from episodic antiretroviral exposure, maternal morbidity and mortality, and sexual transmission [1].

Data from earlier PMTCT eras when women were offered prophylactic antiretrovirals to reduce perinatal transmission risks highlight adherence challenges during pregnant and postpartum periods [2, 3, 4, 5, 6]. Data suggested that taking ART to support their child *and* their own health may promote improved adherence for women [7]. More recent data after institution of Option B+, however, show low rates of viral suppression and high losses to follow‐up: for example in eSwatini 53% postpartum retention in care [8], in Tanzania 39% viral suppression at 12 months postpartum [9]. Follow‐up studies with tracing of women lost to specific clinic follow‐up show that some women are in care elsewhere, though overall postpartum retention in care estimates remain sub‐optimal [10]. For women retained in care, adherence to therapy remains a challenge. In Mpumalanga Province, South Africa, self‐reported adherence to ART was 69% among pregnant WLWH [11] and in Zimbabwe, only 40% of women were taking at least 95% of ART by pill count one year postpartum [12]. Qualitative data from sub‐Saharan African sites suggest that adherence challenges for pregnant and postpartum WLWH relate to structural barriers to care, community and healthcare stigma, medication side effects, depression, substance use and the overwhelming challenges of poverty, gender norms, and life with a newborn [13, 14, 15, 16, 17, 18, 19, 20].

To better understand adherence patterns relative to pregnancy among women initiating ART in rural, southwestern Uganda and urban South Africa in the Option B+ era, we evaluated daily ART adherence over the first year of treatment in a cohort with low losses to follow‐up. We evaluated the impact of pregnancy and postpartum follow‐up and associated factors on daily objectively measured adherence and viral suppression in two diverse African settings. Findings may inform interventions to maximize health and reproductive choice for women, infants and families.

## 2 METHODS

### 2.1 Study settings

In Uganda, participants were recruited from five health centres in a rural region of southwestern Uganda. Clinics included a tertiary care referral site, as well as outpatient clinics providing HIV testing and care. All are located approximately 275 km from Kampala in a largely rural area where HIV prevalence is estimated at 8% for adults and 11% among women accessing antenatal care [21]. Option B+ for PMTCT was implemented in 2013 [22]. Postpartum WLWH are seen together with their infants at four, six, ten and sixteen weeks, and then monthly until 18 months postpartum when women are referred to general HIV care and infants are discharged from the PMTCT clinic.

In South Africa, participants were recruited from three HIV clinics providing integrated HIV care in Gugulethu – an urban township near Cape Town where provincial HIV prevalence is estimated at 13% for adults and 19% for women attending antenatal care [23, 24]. Option B+ for PMTCT was rolled out widely in 2015 [25] and deployed at these sites in 2013 [26]. Per local standard of care, all women were transferred to general ART services after delivery – either after the first routine postpartum visit or when they return for the next ART appointment after delivery.

### 2.2 Participants

Individuals were recruited between March 2015 and September 2016. Inclusion criteria included being ART‐naïve and initiating ART within one month of enrolment date, ≥18 years of age, living within 60 km of the recruitment clinic, and intending to stay in the area for the next year. Enrolled pregnant participants who were at least 34 weeks pregnant per best‐available estimates were excluded (to maximize pregnancy follow‐up time). Inability to provide informed consent was an exclusion criterion.

The parent study enrolled women with CD4 counts <200 cells/mm^3^ and CD4 counts >350 cells/mm^3^ for the primary study question assessing whether initial CD4 cell count (and associated immune function) influences adherence. Pregnant women were only included in the parent study if their CD4 count was at least 350 cells/mm^3^ at enrolment. Therefore, this analysis is limited to women who enrolled with CD4 count at least 350 cells/mm^3^ to reduce selection bias. Results for all participants are published elsewhere [27].

### 2.3 Study procedures

Participants received routine care at their local clinics. Standard first‐line ART (generic tenofovir/emtricitabine/efavirenz in a combination pill) was provided by the study to avoid adherence variations due to formulation or stock outs; changes in ART were made per routine clinical guidance. Participants completed study visits at enrolment, six and twelve months and provided blood for HIV‐1 RNA (determined by the Cobas Taqman Test in Uganda and the Roche CAP/CTM HIV‐1 v2 assay in South Africa) and completed survey questionnaires. Women <50 years of age provided urine for beta‐hCG analysis at each study visit.

Socio‐behavioural factors potentially‐relevant for adherence were chosen based on the Behavioral Model for Vulnerable Populations [28] and included socio‐demographics, structural barriers to care (13‐item scale, scored 0 to 52, higher scores indicate more barriers [29]), food insecurity (9‐item Household Food Insecurity Access Scale [30], scored 0 to 27), HIV stigma (12 items adapted from the Berger HIV Stigma Scale [31] exploring perceived negative attitudes and disclosure concerns, total scoring range 1 to 4, higher score indicating more stigma), HIV disclosure, coping (7 item scale, scoring range 1 to 4, higher score indicating more maladaptive coping [32]), medical mistrust/conspiracy (8 item scale, scoring range 1 to 4, higher score indicating more mistrust/conspiracy [33]), necessity and concerns about taking ART (19 item scale [34]), clinic satisfaction (19 item scale, scoring range 1 to 4, higher score indicating more satisfaction [35]), mental and physical health (MOS‐HIV, 35 items, scored 0 to 100, normalized with 50 indicating average health in a US population [36]), Hopkins Symptom Checklist (HSCL) to screen for anxiety and depression (25 items, each rated 1 to 4, average score of ≥1.75 indicative of high‐risk for psychological distress [37, 38, 39], Edinburg Postnatal Depression Score (EPDS) (10 item score, range 0 to 30 with score of ≥13 indicating positive screen for depression) [37, 40] and high‐risk alcohol use (AUDIT‐C [41, 42]). The degree of emotional or instrumental support (described to participants in general terms for their own interpretation) received from social network ties was assessed by using name generator questionnaires to elicit up to 20 ties, each of whom were rated for the degree of support provided to the participant [43]. The social support score reflects the proportion of each participant’s supporters who were categorized as completely supportive. Adherence was monitored with a real‐time electronic adherence monitor that transmits a date‐and‐time stamp via cellular networks for all opening events as a proxy for medication ingestion (Wisepill Technologies, Cape Town, South Africa).

### 2.4 Analysis

#### 2.4.1 Descriptive statistics

Participant characteristics were summarized descriptively by study site and ever‐pregnant status. Comparisons between sites were made with Wilcoxon rank sum and Fisher’s exact test for continuous and categorical variables respectively.

##### Primary predictor

The primary predictor of interest was women’s pregnancy status over 12‐month follow‐up. Follow‐up time for each woman was categorized as pregnant, postpartum or non‐pregnancy‐related. Women with positive beta‐hCG were categorized as pregnant. Women reporting a birth outcome with a date were categorized as postpartum from that date, including for pre‐term pregnancy losses. Women who were no longer pregnant by beta‐hCG testing and did not have a birth outcome reported were counted as postpartum from 40 weeks after their last menstrual period or to include a total pregnancy length of approximately 40 weeks. Once classified as postpartum, women remained postpartum through the rest of the study (no repeat pregnancies were observed). Median postpartum follow‐up time was 7.4 months (interquartile range [IQR], 5.5 to 8.8) in Uganda and 9.2 months (IQR, 7.6 to 11.3) in South Africa. Women without pregnancy detected or reported during follow‐up contributed to non‐pregnancy‐related time.

To test our hypothesis that adherence may differ by pregnant, postpartum and non‐pregnancy‐related status, we fitted fractional regression models with cluster‐correlated robust estimates of variance to the pooled data [44]. ART adherence was specified as a proportion ranging from 0 to 1 inclusively. For the conditional mean model, we specified a logistic model and made no assumptions about the distribution of the model’s unobserved components. Covariate selection was guided by the Theory of Vulnerable Populations [28]. In the event that the theoretical model suggested multiple potentially collinear variables (e.g. food insecurity and socio‐economic status), we selected covariates that were most relevant based on the enrolment data. Data from women in Uganda and South Africa were analysed separately because of baseline socio‐economic and cultural differences which could affect adherence (Table 1). To facilitate interpretation, the estimated regression coefficients were converted to average marginal effects, or a change in adherence corresponding to a unit change in the explanatory variable of interest, with the values of all other explanatory variables as is. For continuous variables (e.g. age) the average marginal effect corresponds to the first derivative of the response with respect to the variable in question. For discrete variables (e.g. pregnancy status), the average marginal effect corresponds to a discrete change effect and is interpreted relative to the reference group; in effect, this calculation compares two hypothetical populations that have same values for the other explanatory variables in the model.

**Table 1:** Key enrolment demographics and socio‐behavioural factors informed by the Behavioral Model for Vulnerable Populations by site and never/ever pregnant status

Characteristic	Uganda Mean (SD) or N (%)				South Africa Mean (SD) or N (%)			*p* value for all Ugandan vs. all SA women
	All (N = 247)	Never pregnant (N = 99)		Ever pregnant (N = 148)	All (N = 190)	Never pregnant (N = 108)	Ever pregnant (N = 82)	
Demographics								
Age	29 (9)	32 (11)		26 (5)	31 (9)	35 (10)	27 (5)	0.001*
Pregnant at enrolment	*129 (52)*	*–*			*76 (40)*	*–*		0.012*
Incident pregnancy during follow‐up	*19 (16)*	*–*			*6 (5)*	*–*		0.01*
Married	*143 (59)*	*34 (34)*		*109 (76)*	*29 (15)*	*22 (20)*	*7 (9)*	<0.001*
Highest education								<0.001*
None/primary	*84 (35)*	*42 (42)*		*42 (29)*	*14 (7)*	*9 (8)*	*5 (6)*	
Secondary	*159 (65)*	*57 (58)*		*102 (71)*	*175 (93)*	*99 (92)*	*76 (94)*	
Employed	*187 (77)*	*86 (87)*		*101 (70)*	*75 (40)*	*49 (45)*	*26 (32)*	<0.001*
Structural barrier score	29 (12)	3.5 (5.6)		2.7 (5.6)	82 (43)	13.3 (7.1)	15.0 (7.2)	<0.001*
Health								
Median CD4 count (cells/mL)	477 (421, 618)	441 (399, 485)		554 (438, 676)	440 (394, 490)	426 (386, 462)	477 (413, 662)	<0.001*
Median HIV‐RNA (log_10_ c/mL)	3.6 (2.5, 4.3)	3.6 (2.6, 4.4)		3.6 (2.2, 4.3)	4.2 (3.6, 4.6)	4.2 (3.6, 4.8)	4.2 (3.4, 4.5)	<0.001*
HIV dx greater than 30 days prior to enrolment	221 (91)	96 (97)		125 (87)	138 (79)	96 (99)	42 (55)	<0.001*
Median days from diagnosis to enrolment	59 (4, 874)	285 (8, 1808)		34 (2.5, 420.5)	40.5 (2, 730)	73 (22, 797)	1 (0, 383)	0.286
Mental wellbeing	48 (11)	47 (11)		48 (11)	38 (9)	39 (9)	38 (8)	0.001*
Physical wellbeing	41 (5)	41 (4)		41 (5)	44 (8)	44 (8)	45 (7)	<0.001*
Depressed (>9 on EPDS)	*55 (23)*	*24 (24)*		*31 (22)*	*105 (56)*	*65 (60)*	*40 (49)*	<0.001*
Coping score		2.2 (0.4)		2.2 (0.4)		2.4 (0.3)	2.3 (0.2)	
Severe food insecurity	*69 (28)*	*32 (32)*		*37 (26)*	*121 (64)*	*68 (63)*	*53 (65)*	<0.001*
Heavy alcohol use	*19 (8)*	*10 (10)*		*9 (6)*	*47 (25)*	*30 (28)*	*17 (21)*	<0.001*
Medications other than ART	*207 (85)*	*90 (91)*		*117 (81)*	*25 (13)*	*22 (20)*	*3 (4)*	<0.001*
Cigarette use	*5 (2)*	*3 (3)*		*2 (1)*	*27 (14)*	*16 (15)*	*11 (14)*	<0.001*
Reproductive health history								
# of biological children	2 (0, 3)		2 (1, 4)	1 (0, 2)	1 (1, 2)	2 (1, 3)	1 (1, 2)	0.27
Estimated GA at enrolment, weeks (limited to those enrolling pregnant)	*–*		*–*	19 (12, 24)	*–*	*–*	24 (19, 28)	<0.001*
Partner/relationships								
Relationship w/main partner								
Spouse	*149 (69)*		*37 (49)*	*112 (81)*	*45 (27)*	*30 (33)*	*15 (19)*	<0.001*
Regular	*55 (26)*		*33 (43)*	*22 (16)*	*118 (69)*	*55 (61)*	*63 (79)*	
Other	*11 (5)*		*6 (8)*	*5 (4)*	*7 (4)*	*5 (6)*	*2 (2)*	
Main partner HIV serostatus								0.055
Negative	*27 (13)*		*8 (11)*	*19 (14)*	*22 (13)*	*14 (15)*	*8 (10)*	
Positive	*78 (36)*		*30 (39)*	*48 (34)*	*44 (25)*	*31 (33)*	*13 (16)*	
Don’t know	*110 (51)*		*38 (50)*	*72 (52)*	*108 (57)*	*49 (52)*	*58 (73)*	
Refused			*–*‐	*–*‐		0 (0)	1 (1)	
Physical violence past six months	*31 (18)*		*12 (19)*	*19 (17)*	*6 (3)*	*3 (3)*	*3 (4)*	<0.001*
Exchange money for sex past six months	*33 (14)*		*11 (11)*	*22 (15)*	*3 (2)*	*1 (1)*	*2 (3)*	<0.001*
Forced sex past six months	*33 (14)*		*11 (12)*	*22 (15)*	*9 (5)*	*7 (7)*	*2 (3)*	0.002*
Disclosure/stigma/social support/relationship with HC system								
Disclosed aside from healthcare worker	*188 (77)*		*79 (80)*	*109 (76)*	*137 (73)*	*92 (85)*	*45 (56)*	0.24
Disclosed outside of household	*123 (51)*		*60 (61)*	*63 (44)*	*74 (39)*	*55 (51)*	*19 (24)*	0.025*
Stigma: perceived negative attitudes towards HIV	1 (0, 4)		1 (0, 3)	2 (0, 4)	3 (0, 4)	3 (1, 4)	2 (0, 4)	0.032*
Stigma: disclosure concerns	5 (2, 6)		4 (1, 6)	5 (2, 7)	3 (1, 6)	3 (1, 5.5)	4 (1, 6)	0.06
Instrumental support	0.4 (0.2)		0.4 (0.2)	0.4 (0.2)	0.5 (0.3)	0.5 (0.3)	0.6 (0.3)	<0.001*
Emotional support	0.5 (0.3)		0.5 (0.3)	0.6 (0.3)	0.8 (0.3)	0.7 (0.3)	0.6 (0.3)	<0.001*
Healthcare system/medication trust								
Medical mistrust	1.75 (1, 2.5)		1.75 (1, 2.25)	1.75 (1, 2.5)	3 (2, 2.5)	2 (2, 2.5)	2 (2, 2.5)	<0.001*
Conspiracy	1.9 (0.7)		1.9 (0.7)	1.9 (0.7)	2.3 (2, 2.5)	2.3 (2, 2.7)	2.3 (2, 2.5)	<0.001*
Clinic satisfaction	3.5 (0.4)		3.5 (0.4)	3.5 (0.4)	3.1 (0.4)	3.1 (0.4)	3.1 (0.4)	<0.001*
ART need								
Low	*40 (16)*		*17 (17)*	*23 (16)*	*87 (46)*	*46 (43)*	*41 (51)*	<0.001*
Moderate	*94 (39)*		*40 (40)*	*54 (38)*	*81 (43)*	*50 (46)*	*31 (38)*	
High	*109 (45)*		*42 (42)*	*67 (47)*	*21 (11)*	*12 (11)*	*9 (11)*	
ART concerns								
Low	*46 (19)*		*11 (11)*	*35 (24)*	*8 (4)*	*4 (4)*	*4 (5)*	<0.001*
Moderate	*74 (31)*		*41 (41)*	*33 (23)*	*67 (36)*	*37 (34)*	*30 (37)*	
High	*123 (51)*		*47 (48)*	*76 (53)*	*114 (60)*	*67 (62)*	*47 (58)*	

All *p*‐values determined by Fishers exact test for categorical and Wilxocon rank sum for continuous variables between all Uganda women and all South Africa women. Values in italics are dichotomous variables listed as N (%). Remaining variables are continuous and either listed as mean (SD) or median (IQR).

* Significant at *p* < 0.05 level.

#### 2.4.2 Outcomes

##### Adherence

Adherence was calculated as the number of electronic adherence monitor opening events observed divided by the number of opening events expected during follow‐up. Adherence was capped at 100% per day and censored at death. Adherence for participants lost to follow‐up (LTFU), defined as no contact despite multiple attempts through 13 months after enrolment, was calculated until the device stopped transmitting data or until the last scheduled study visit – whichever came first. Monitor opening events related to staff openings and during periods of known device malfunction were removed from the data [45].

Adherence interruptions of seven or more days were also assessed [46]. Adherence distributions between groups of never‐ and ever‐pregnant women were compared by the Wilcoxon rank sum test.

To compare adherence before and after the expected due date, first we visually inspected the distribution of adherence over time. To formally assess the difference in median adherence before vs. after the expected due date, we included an interaction term in the regression models.

In sensitivity analyses, we imputed adherence data for women LTFU as either [1] mean adherence prior to loss to follow‐up or [2] mean adherence for postpartum women with adherence data. These means were compared with the primary (non‐imputed) results via Student’s t‐test.

##### HIV‐RNA suppression

Viral suppression was defined as <400 copies/mL. Participants were defined to be viraemic if they died (any cause). Those LTFU or who had missed visits and had <80% adherence in the month prior to the missed visit were counted as viraemic.

All analyses were conducted in Stata 13 (StataCorp., College Station, TX, USA).

#### 2.4.3 Ethics

This study was approved by the human research ethics committees of Partners Healthcare, Mbarara University of Science and Technology, and the University of Cape Town. Additional regulatory approvals were secured from the Uganda National Council for Science and Technology and the Western Cape province in South Africa.

## 3 RESULTS

### 3.1 Demographics

This analysis includes 247 women in Uganda and 190 women in South Africa. In Uganda, 129 (39%) women were pregnant at enrolment and an additional 19 (6%) women had pregnancies over the course of follow‐up. Participants in Uganda contributed 676 months of pregnancy follow‐up time, 900 months of postpartum follow‐up time and 1274 months of non‐pregnancy‐related follow‐up time. At enrolment, Ugandan women had a mean age of 29 years (standard deviation (SD), 9); 59% were married. Most (77%) reported formal or informal employment with 28% reporting severe food insecurity but a low structural barrier score (median 0). Median CD4 cell count was 477 cells/mm^3^ (IQR, 421 to 618) and most (91%) reported being diagnosed with HIV at least one month prior to enrolment. Thirty‐six percent reported a main partner living with HIV; 51% did not know their partner’s serostatus. Fifty‐five (23%) met screening criteria for depression by HSCL, 30 (30%) met screening criteria by EPDS and 19 (8%) reported heavy alcohol use. (Table 1).

In South Africa, 76 (26%) women were pregnant at enrolment and an additional 6 (2%) women had pregnancies during follow‐up. Participants contributed 259 months of pregnancy follow‐up time, 624 months of postpartum follow‐up time and 1247 months of non‐pregnancy‐related‐follow‐up time. At enrolment, participants had a median age of 31 years (SD, 9); 15% were married. Few (40%) reported formal or informal employment with 64% reporting severe food insecurity and a high structural barrier score (median 13; IQR, 8 to 18). Median CD4 cell count was 440 cells/mm^3^ (IQR, 394 to 490) and 79% reported being diagnosed with HIV at least one month prior to enrolment. Most (61%) did not know their partner’s serostatus. Over half (56%) met the screening criteria for depression by HSCL, 30 (40%) met screening criteria by EPDS and 47 (25%) reported heavy alcohol use. Ever‐pregnant women in South Africa were younger and more likely to have been diagnosed with HIV in the last 30 days. (Table 1).

A total of 39/437 (9%) women across both sites (15/247, 6% in Uganda and 24/190, 13% in South Africa) had incomplete data including those who were LTFU (7 in Uganda and 13 in SA), disenrolled (4 in Uganda and 11 in SA) or died (4 in Uganda). Among these 39 women, 16 women (4 in Uganda and 12 in SA) contributed no adherence data; 23 women (11 in Uganda and 12 in SA) maintained Wisepill devices contributing adherence data for a mean of 78 (SD, 97) days. For 14 women (4 in Uganda, 10 in South Africa) viral load data were missing.

### 3.2 Adherence

#### 3.2.1 Uganda

**Figure 1:**
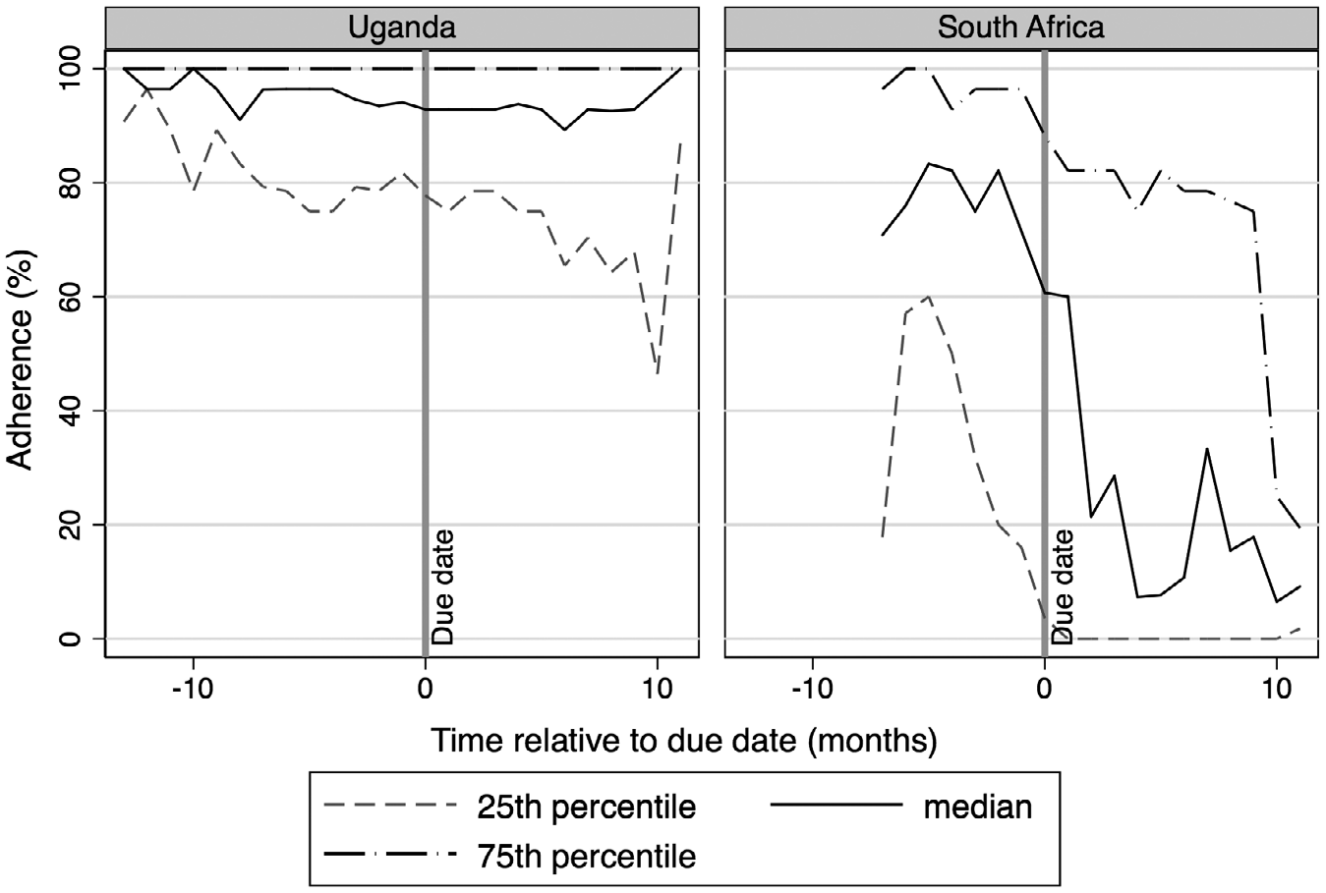
Average (IQR) adherence relative to due date among pregnant women living with HIV and accessing ART in Uganda and South Africa (Only women with pregnancy included in this analysis). Change in slope for median and 25th percentile statistically significant (*p* < 0.001) for Uganda cohort. All slopes statistically significant (*p* < 0.001) for the South Africa cohort.

In Uganda, median adherence to ART for women during pregnancy, postpartum and non‐pregnancy‐related periods was 94% (IQR, 78% to 98%), 90% (IQR, 70% to 97%), 90% (IQR, 80% to 98%) (*p* = 0.017). Figure 1 plots adherence relative to pregnancy period follow‐up and estimated due date, demonstrating a downward trend in median adherence after due date (*p* < 0.001). Seven‐day interruptions were rare for all women (median, 0; IQR, 0 to 1). Imputing adherence for those women LTFU as either the mean for their adherence prior to loss to follow‐up group or the mean for postpartum women LTFU but with adherence data, did not result in statistically different adherence estimates. Mean adherence was independently associated with age (average 0.98% increase in adherence per year of age; 95% CI, 0.33% to 1.62%) and CD4 cell count (average 1.01% decrease per 50 cells/mm^3^ increase in CD4 count; 95% CI, 0.08% to 1.94%) (Table 2).

**Table 2:** Correlates of ART adherence as measured with daily monitoring of pill cap openings among women in Uganda (a) and South Africa (b)

Covariate	Average marginal effect	95% CI
a. Uganda		
Non‐pregnancy‐related follow‐up	*Ref*	*Ref*
Pregnancy follow‐up	0.0617	−0.0079, 0.1313
Postpartum follow‐up	0.0074	−0.0704, 0.0853
**Age**	**0.0098**	**0.0033, 0.0162**
**CD4 cell count (per 50 cells)**	−**0.0101**	−**0.0194,** −**0.0008**
Highest education – secondary	0.0104	−0.0489, 0.0696
Structural barrier score	−0.0020	−0.0082, 0.0042
Perceived stigma	−0.0145	−0.0313, 0.0023
Severe food insecurity	−0.0425	−0.1128, 0.0277
Depressed (EPDS > 9)	0.0254	−0.0614, 0.1122
Alcohol abuse (AUDIT‐C)	−0.0499	−0.1539, 0.0540
Instrumental support	0.0033	−0.0018, 0.0085
Emotional support	−0.0013	−0.0059, 0.0033
Main sexual partner relationship	0.0038	−0.1000, 0.0925
b. South Africa		
Non‐pregnancy‐related follow‐up	*Ref*.	*Ref*.
Pregnancy follow‐up	0.0512	−0.0766, 0.1791
**Postpartum follow‐up**	−**0.2229**	−**0.3540,** −**0.0859**
Age	0.0028	−0.0054, 0.0110
CD4 count (per 50 cells)	0.0113	−0.0094, 0.0340
Highest education – secondary	−0.0276	−0.2180, 0.1628
Structural barrier score	0.0039	−0.0044, 0.0121
Perceived stigma	0.0154	−0.0177, 0.0484
Severe food insecurity	−0.0389	−0.1648, 0.0869
Depressed (EPDS > 9)	−0.0022	−0.1195, 0.1150
Alcohol abuse (AUDIT‐C)	−0.0743	−0.2139, 0.0653
Instrumental support	−0.0093	−0.0215, 0.0029
**Emotional support**	**0.0140**	**0.0022, 0.0258**
Main sexual partner relationship	0.0126	−0.1360, 1612

Boldface type indicates statistical significance at the *p* < 0.05 level. The joint *p*‐value for the adjusted association between pregnancy status and adherence is 0.0102 for the Ugandan sample and <0.0001 for the South African sample. Estimated regression coefficients have been converted to average marginal effects for ease of exposition.

**Figure 2:**
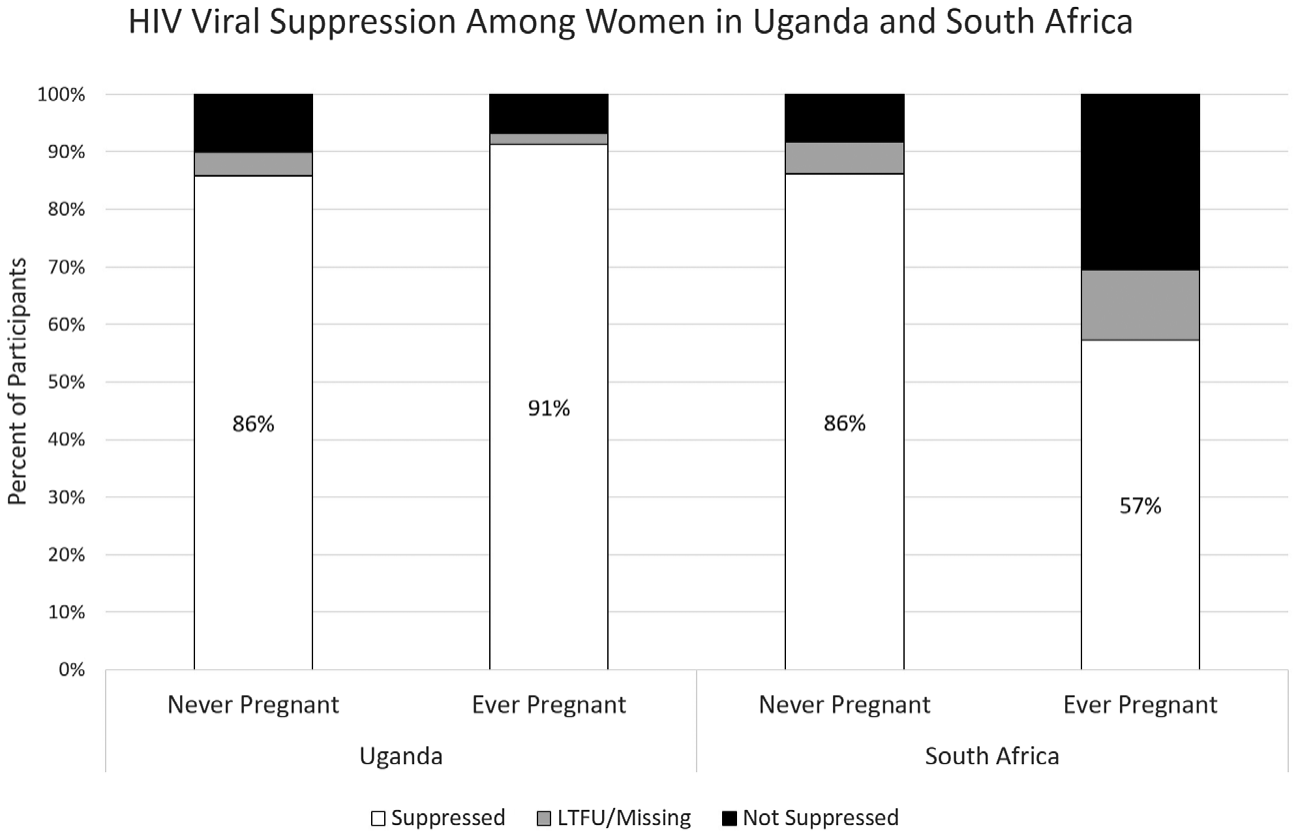
Percentage of women with HIV‐RNA suppression at 12 months by site and ever‐pregnant vs. never‐pregnant status

In Uganda, 135 (91%) and 85 (86%) of ever‐ and never‐pregnant women achieved HIV‐RNA suppression at 12 months of follow‐up. If we assume that all women LTFU or otherwise missing were in care and suppressed, then 138/148 (93%) ever pregnant women and 89/99 (89%) never‐pregnant women were virally suppressed at one‐year follow‐up (Figure 2).

#### 3.2.2 South Africa

Among South African participants, median ART adherence was 74% during pregnant periods (IQR, 31% to 96%), 40% during postpartum (IQR, 4% to 65%) and 77% during non‐pregnancy periods (IQR, 47% to 92%) (*p* < 0.001). Daily median adherence relative to pregnancy period follow‐up and estimated due date demonstrates a downward trend after due date (*p* < 0.001) (Figure 1). The median (IQR) number of seven‐day interruptions was 0 during pregnant periods (IQR, 0 to 0.6), 0.4 during postpartum (IQR, 0.1 to 0.7) and 0.2 during non‐pregnancy periods (IQR, 0 to 0.4). Imputing adherence for women LTFU as either the mean for their adherence prior to loss to follow‐up or the mean for postpartum women LTFU but with adherence data did not result in statistically different adherence estimates. Mean adherence was associated with postpartum follow‐up (average 22.3% lower adherence during postpartum compared with non‐pregnancy‐related follow‐up; 95% CI, 8.6% to 35.4%) and emotional support (average 1.4% higher adherence per unit of increased emotional support; 95% CI, 0.22% to 2.58%).

HIV‐RNA suppression was achieved by 47 (57%) and 93 (86%) of ever‐ and never‐pregnant women (Figure 2). If we assume that those LTFU or missing HIV‐RNA data were in care elsewhere, then up to 57 (69%) ever‐pregnant women and 99 (92%) never‐pregnant women were virally suppressed (Figure 2).

## 4 DISCUSSION

In this observational study capturing daily adherence behaviour among women enrolled with CD4 counts of ≥350 cells/mm^3^, postpartum status was independently associated with worse adherence compared to non‐pregnant status in South Africa. Postpartum women in South Africa adhered to a median of 40% of daily ART doses and only 57% of women with a pregnancy achieved HIV‐RNA suppression one year after starting ART. In adjusted models, emotional support independently affected adherence. We also observed preservation of adherence and 91% one‐year viral suppression among women with pregnancy in rural Uganda. Importantly, there were low losses to follow‐up and medications were provided by the study, highlighting challenges with daily pill taking even when women remain in care. These data indicate the importance of local context and an urgent situation in South Africa with poor adherence leading to viremia in pregnant and postpartum women.

Policy makers, clinicians and advocates hoped that simpler regimens and messages emphasizing the importance of ART to women’s health would improve adherence and HIV outcomes for women. Our findings indicate ongoing challenges, particularly in the South African cohort, during the postpartum period. Daily adherence data support earlier observations: the postpartum period is fraught with challenges and serves as a “cliff” in HIV care adherence [16]. In the current era where most women living with HIV access ART, at least half of new infections occur during breastfeeding – including in South Africa and Uganda where nearly all pregnant WLWH access ART [47]. While adherence counselling through pregnancy tends to focus on the health of the child, and women may focus on the goal of delivering an HIV‐uninfected child, postpartum messaging may be less clear, particularly when women do not receive postpartum‐specific HIV care. Yet viremia postpartum has dire consequences for breastfeeding infants.

Qualitative data published from this cohort described pregnancy and the postpartum state as “de‐stabilizing experiences” in which multiple factors including changes in the relationship with the father of the child (leading to poverty and lack of emotional support), inability to work and provide for oneself and physical symptoms compromise adherence [48]. Many women are diagnosed with HIV in pregnancy (nearly half of pregnant women in the South Africa cohort were diagnosed in the month prior to study enrolment); the concomitant stigma of living with HIV and having a pregnancy may further complicate medication adherence [49, 50, 51, 52, 53, 54]. Postpartum, acute worries around delivering a healthy infant may resolve after birth and change motivations for remaining on treatment or in care [14, 15]. The stress of caring for a newborn can be overwhelming, leaving little time for self‐care. In South Africa, women are generally transferred from obstetric care to another clinic upon delivery, resulting in potential for losses to follow‐up, confusion, logistical challenges, and/or suboptimal support [17] [26].

Nine percent of women were LTFU, disenrolled or died. We were unable to trace all women and therefore some of those LTFU may have been retained in care. However, even if all women LTFU had remained in care and achieved HIV‐RNA suppression, we would still observe low rates of viral suppression among women in South Africa. Retention in HIV care remains an important issue for pregnant and postpartum women living with HIV [55, 56]. In the Ugandan sites, where adherence was high and losses to follow‐up minimal, women live in rural settings where most farm and are relatively non‐mobile. In addition, WLWH remain in mother‐baby coupled clinical care until 18 months postpartum.

In South Africa, the only factor independently associated with adherence in addition to postpartum follow‐up was emotional support which may be crucial to helping women overcome structural barriers to access care or physical challenges associated with being postpartum [48]. Details on the types of emotional support provided were not assessed in survey data, but in qualitative data, participants described the importance of reminders and encouragement to take their medication from social supports [48]. Among women in Uganda, older age and lower baseline CD4 cell count were associated with improved adherence. Older age is a factor frequently associated with greater ART adherence perhaps related to health literacy and life stability [57, 58], although this observation largely focuses on those ages >50 years; the age range of this study was limited to those age ≤50. Studies of women taking HIV pre‐exposure prophylaxis (PrEP) observe improved adherence with increasing age in younger cohorts [59], perhaps related to neurodevelopmental factors [60]. A combination of both factors may be relevant in this cohort of relatively young women. Some have postulated that a lower baseline CD4 cell count and poorer health could prompt increased motivations to adhere to therapy [61, 62], which may be relevant here. That said, all of the women in this analysis initiated therapy with a relatively preserved CD4 cell count (i.e. ≥350 cells/mm^3^).

Some interventions to support women to transition from PMTCT to general ART care and to retain postpartum women in ART programmes will promote emotional support in ongoing studies with peer navigators/mentor mothers [63, 64] as well as increased male involvement [65]. In addition, studies addressing structural factors showed promising results with integration of postpartum care into routine care for the child or family [66] [67], mHealth reminders and cash transfers [68, 69, 70, 71, 72]. Ongoing studies will evaluate effects of mHealth strategies [73, 74], reaching women with mental health challenges in pregnancy and postpartum [75, 76] and adherence clubs/decentralized care [77]. Women in different contexts will require different types of support [78, 79] and an adaptive trial design [80] may be an efficient way to evaluate strategies to combine intervention elements to support women to remain in care [81].

This observational study has limitations. We did not follow‐up infants for perinatal transmission outcomes. Additionally, there is potential for device non‐use to influence adherence data resulting in misclassification bias. Notably, previous studies have found to be moderate correlation between electronic monitoring and drug concentration in blood [82, 83], and the HIV‐RNA data in this study align with the adherence findings. Strengths include inclusion of women from urban South Africa and rural Uganda with longitudinal objective adherence measurement, HIV‐RNA, minimal losses to follow‐up and models including behavioural factors that influence adherence based on a conceptual framework.

## 5 CONCLUSIONS

Many WLWH struggle to remain in care and on ART during the postpartum period. For their own health and that of their infants, effective, context‐specific interventions are needed to support women in this critical period. These data suggest that interventions to provide emotional support during the postpartum period for younger women may be important.

## References

[1] World Health Organization . Consolidated guidelines on the use of antiretroviral drugs for treating and preventing HIV infection: summary of key features and recommendations, June 2013. 2013. 24716260

[2] Nachega JB , Uthman OA , Anderson J , Peltzer K , Wampold S , Cotton MF , et al. Adherence to antiretroviral therapy during and after pregnancy in low‐income, middle‐income, and high‐income countries: a systematic review and meta‐analysis. Aids. 2012;26(16):2039–52. 22951634 10.1097/QAD.0b013e328359590f PMC5061936

[3] Sha BE , Tierney C , Cohn SE , Sun X , Coombs RW , Frenkel LM , et al. Postpartum viral load rebound in HIV‐1‐infected women treated with highly active antiretroviral therapy: AIDS Clinical Trials Group Protocol A5150. HIV Clin Trials. 2011;12(1):9–23. 21388937 10.1310/hct1201-9 PMC3227722

[4] Kilewo C , Karlsson K , Ngarina M , Massawe A , Lyamuya E , Swai A , et al. Prevention of mother‐to‐child transmission of HIV‐1 through breastfeeding by treating mothers with triple antiretroviral therapy in Dar es Salaam, Tanzania: the Mitra Plus study. J Acquir Immune Defic Syndr. 2009;52(3):406–16. 19730269 10.1097/QAI.0b013e3181b323ff

[5] Ngarina M , Popenoe R , Kilewo C , Biberfeld G , Ekstrom AM . Reasons for poor adherence to antiretroviral therapy postnatally in HIV‐1 infected women treated for their own health: experiences from the Mitra Plus study in Tanzania. BMC Public Health. 2013;13(1):450. 23647555 10.1186/1471-2458-13-450 PMC3651864

[6] Onoya D , Sineke T , Brennan AT , Long L , Fox MP . Timing of pregnancy, postpartum risk of virologic failure and loss to follow‐up among HIV‐positive women. Aids. 2017;31(11):1593–602. 28463877 10.1097/QAD.0000000000001517 PMC5491237

[7] Cavallo IK , Kakehasi FM , Andrade BA , Lobato AC , Aguiar RA , Pinto JA , et al. Predictors of postpartum viral load rebound in a cohort of HIV‐infected Brazilian women. Int J Gynaecol Obstet. 2010;108(2):111–4. 19892340 10.1016/j.ijgo.2009.09.014

[8] Abrams EJ , Langwenya N , Gachuhi A , Zerbe A , Nuwagaba‐Biribonwoha H , Mthethwa‐Hleta S , et al. Impact of universal ART for pregnant and postpartum women on ART uptake and retention. AIDS. 2019;33(1):45–4. 30289804 10.1097/QAD.0000000000002027

[9] Ngarina M , Kilewo C , Karlsson K , Aboud S , Karlsson A , Marrone G , et al. Virologic and immunologic failure, drug resistance and mortality during the first 24 months postpartum among HIV‐infected women initiated on antiretroviral therapy for life in the Mitra plus Study, Dar es Salaam, Tanzania. BMC Infect Dis. 2015;15(1):175. 25886277 10.1186/s12879-015-0914-z PMC4392730

[10] Reidy W , Nuwagaba‐Biribonwoha H , Shongwe S , Sahabo R , Hartsough K , Wu Y , et al. Engagement in care among women and their infants lost to follow‐up under Option B+ in eSwatini. PLoS One. 2019;14:e0222959. 31665137 10.1371/journal.pone.0222959 PMC6821080

[11] Ramlagan S , Peltzer K , Ruiter RAC , Barylski NA , Weiss SM , Sifunda S . Prevalence and factors associated with fixed‐dose combination antiretroviral drugs adherence among HIV‐positive pregnant women on option B treatment in mpumalanga province, South Africa. Int J Environ Res Public Health. 2018;15(1):161. 10.3390/ijerph15010161 PMC5800260 29361675

[12] Erlwanger AS , Joseph J , Gotora T , Muzunze B , Orne‐Gliemann J , Mukungunugwa S , et al. Patterns of HIV Care clinic attendance and adherence to antiretroviral therapy among pregnant and breastfeeding women living with HIV in the context of option B+ in Zimbabwe. J Acquir Immune Defic Syndr. 2017;75 Suppl 2:S198–206. 28498190 10.1097/QAI.0000000000001347

[13] Gill MM , Umutoni A , Hoffman HJ , Ndatimana D , Ndayisaba GF , Kibitenga S , et al. Understanding antiretroviral treatment adherence among HIV‐positive women at four postpartum time intervals: qualitative results from the Kabeho Study in Rwanda. AIDS Patient Care STDS. 2017;31(4):153–66. 28358624 10.1089/apc.2016.0234

[14] Ashaba S , Kaida A , Burns BF , O'Neil K , Dunkley E , Psaros C , et al. Understanding coping strategies during pregnancy and the postpartum period: a qualitative study of women living with HIV in rural Uganda. BMC Pregnancy Childbirth. 2017;17(1):138. 28482821 10.1186/s12884-017-1321-9 PMC5423027

[15] Ashaba S , Kaida A , Coleman JN , Burns BF , Dunkley E , O'Neil K , et al. Psychosocial challenges facing women living with HIV during the perinatal period in rural Uganda. PLoS One. 2017;12:e0176256. 28459866 10.1371/journal.pone.0176256 PMC5411062

[16] Psaros C , Remmert JE , Bangsberg DR , Safren SA , Smit JA . Adherence to HIV care after pregnancy among women in sub‐Saharan Africa: falling off the cliff of the treatment cascade. Curr HIV/AIDS Rep. 2015;12(1):1–5. 25620530 10.1007/s11904-014-0252-6 PMC4370783

[17] Pellowski JA , Weber AZ , Phillips TK , Brittain K , Zerbe A , Abrams EJ , et al. “You must leave but I didn’t want to leave”: qualitative evaluation of the integration of ART into postnatal maternal and child health services in Cape Town, South Africa. AIDS Care. 2020;32(4):480–5. 31455090 10.1080/09540121.2019.1659913 PMC7044018

[18] Phillips T , McNairy ML , Zerbe A , Myer L , Abrams EJ . Implementation and operational research: postpartum transfer of care among HIV‐infected women initiating antiretroviral therapy during pregnancy. J Acquir Immune Defic Syndr. 2015;70(3):e102–9. 10.1097/QAI.0000000000000771 26470033

[19] Raggio GA , Psaros C , Fatch R , Goodman G , Matthews LT , Magidson JF , et al. High rates of biomarker‐confirmed alcohol use among pregnant women living with HIV in South Africa and Uganda. J Acquir Immune Defic Syndr. 2019;82(5):443–51. 31567551 10.1097/QAI.0000000000002156 PMC6857734

[20] Rotheram‐Borus MJ , Weichle TW , Wynn A , Almirol E , Davis E , Stewart J , et al. Alcohol, but not depression or IPV, reduces HIV adherence among south african mothers living with HIV over 5 years. AIDS Behav. 2019;23(12):3247–56. 31401739 10.1007/s10461-019-02617-2 PMC6854305

[21] Uganda Ministry of Health IC . Uganda Population Based HIV Impact Assessment (UPHIA): 2016–2017. 2017 [cited 2019 Dec 10]. Available from: https://phia.icap.columbia.edu/wp-content/uploads/2018/07/3430%E2%80%A2PHIA-Uganda-SS_NEW.v14.pdf

[22] Ministry of Health Uganda . Addendum to the Antiretroviral Treatment Guidelines for Uganda. 2013. Report No.

[23] South Africa National Department of Health . The 2015 National Antenatal Sentinel HIV and Syphilis Survey, South Africa. 2017.

[24] South Africa National Department of Health, Human Sciences Research Council . The fifth South African national HIV prevalence, incidence, behaviour and communication survey, 2017 (SABSSM V1). Pretoria. 2019

[25] Republic of South Africa . National Consolidated Guidelines for PMTCT and the Management of HIV in Children, Adolescents and Adults. National Department of Health Republic of South Africa [cited 2019 Dec 10]. Available from: https://sahivsoc.org/Files/ART%20Guidelines%2015052015.pdf2015

[26] Phillips TK , Clouse K , Zerbe A , Orrell C , Abrams EJ , Myer L . Linkage to care, mobility and retention of HIV‐positive postpartum women in antiretroviral therapy services in South Africa. J Int AIDS Soc. 2018;21:e25114. 30027583 10.1002/jia2.25114 PMC6053482

[27] Haberer JE , Bwana BM , Orrell C , Asiimwe S , Amanyire G , Musinguzi N , et al. ART adherence and viral suppression are high among most non‐pregnant individuals with early‐stage, asymptomatic HIV infection: an observational study from Uganda and South Africa. J Int AIDS Soc. 2019;22:e25232. 30746898 10.1002/jia2.25232 PMC6371013

[28] Gelberg L , Andersen RM , Leake BD . The Behavioral Model for Vulnerable Populations: application to medical care use and outcomes for homeless people. Health Serv Res. 2000;34(6):1273–302. 10654830 PMC1089079

[29] Coetzee B , Kagee A . The development of an inventory to assess the structural barriers to clinic attendance and pill‐taking amongst users of antiretroviral therapy. AIDS Behav. 2013;17(1):319–28. 23229338 10.1007/s10461-012-0374-z

[30] Tsai AC , Bangsberg DR , Emenyonu N , Senkungu JK , Martin JN , Weiser SD . The social context of food insecurity among persons living with HIV/AIDS in rural Uganda. Soc Sci Med. 2011;73(12):1717–24. 22019367 10.1016/j.socscimed.2011.09.026 PMC3221802

[31] Jeyaseelan L , Kumar S , Mohanraj R , Rebekah G , Rao D , Manhart LE . Assessing HIV/AIDS stigma in south India: validation and abridgement of the Berger HIV Stigma scale. AIDS Behav. 2013;17(1):434–43. 22246514 10.1007/s10461-011-0128-3 PMC3404245

[32] Mohanraj R , Jeyaseelan V , Kumar S , Mani T , Rao D , Murray KR , et al. Cultural adaptation of the Brief COPE for persons living with HIV/AIDS in southern India. AIDS Behav. 2015;19(2):341–51. 25096895 10.1007/s10461-014-0872-2 PMC4320041

[33] LaVeist TA , Isaac LA , Williams KP . Mistrust of health care organizations is associated with underutilization of health services. Health Serv Res. 2009;44(6):2093–105. 19732170 10.1111/j.1475-6773.2009.01017.x PMC2796316

[34] Horne R , Weinman J . Patients' beliefs about prescribed medicines and their role in adherence to treatment in chronic physical illness. J Psychosom Res. 1999;47(6):555–67. 10661603 10.1016/s0022-3999(99)00057-4

[35] Babikako HM , Neuhauser D , Katamba A , Mupere E . Patient satisfaction, feasibility and reliability of satisfaction questionnaire among patients with pulmonary tuberculosis in urban Uganda: a cross‐sectional study. Health Res Policy Syst. 2011;9:6. 21276274 10.1186/1478-4505-9-6 PMC3042007

[36] Stangl AL , Bunnell R , Wamai N , Masaba H , Mermin J . Measuring quality of life in rural Uganda: reliability and validity of summary scores from the medical outcomes study HIV health survey (MOS‐HIV). Qual Life Res. 2012;21(9):1655–63. 22198741 10.1007/s11136-011-0075-5

[37] Tsai AC , Scott JA , Hung KJ , Zhu JQ , Matthews LT , Psaros C , et al. Reliability and validity of instruments for assessing perinatal depression in African settings: systematic review and meta‐analysis. PLoS One. 2013;8:e82521. 24340036 10.1371/journal.pone.0082521 PMC3858316

[38] Kaaya SF , Fawzi MC , Mbwambo JK , Lee B , Msamanga GI , Fawzi W . Validity of the Hopkins Symptom Checklist‐25 amongst HIV‐positive pregnant women in Tanzania. Acta Psychiatr Scand. 2002;106(1):9–19. 12100343 10.1034/j.1600-0447.2002.01205.x PMC6300056

[39] Ashaba S , Kakuhikire B , Vorechovska D , Perkins JM , Cooper‐Vince CE , Maling S , et al. Reliability, validity, and factor structure of the Hopkins symptom checklist‐25: population‐based study of persons living with HIV in rural Uganda. AIDS Behav. 2018;22(5):1467–74. 28667469 10.1007/s10461-017-1843-1 PMC6613367

[40] Cox JL , Holden JM , Sagovsky R . Detection of postnatal depression. Development of the 10‐item edinburgh postnatal depression scale. Br J Psychiatry. 1987;150:782–6. 3651732 10.1192/bjp.150.6.782

[41] Bush K , Kivlahan DR , McDonell MB , Fihn SD , Bradley KA . The AUDIT alcohol consumption questions (AUDIT‐C): an effective brief screening test for problem drinking. Ambulatory Care Quality Improvement Project (ACQUIP). Alcohol Use Disorders Identification Test. Arch Intern Med. 1998;158(16):1789–95. 9738608 10.1001/archinte.158.16.1789

[42] Bradley KA , Bush KR , Epler AJ , Dobie DJ , Davis TM , Sporleder JL , et al. Two brief alcohol‐screening tests From the Alcohol Use Disorders Identification Test (AUDIT): validation in a female Veterans Affairs patient population. Arch Intern Med. 2003;163(7):821–9. 12695273 10.1001/archinte.163.7.821

[43] Dunkel‐Schetter C , Folkman S , Lazarus RS . Correlates of social support receipt. J Pers Soc Psychol. 1987;53(1):71–80. 3612494 10.1037//0022-3514.53.1.71

[44] www.stata.com . Fractional outcome regression. 2020 [cited 2020 March 1, 2020]. Available from: https://www.stata.com/features/overview/fractional-outcome-models/

[45] Bova CA , Fennie KP , Knafl GJ , Dieckhaus KD , Watrous E , Williams AB . Use of electronic monitoring devices to measure antiretroviral adherence: practical considerations. AIDS Behav. 2005;9(1):103–10. 15812617 10.1007/s10461-005-1685-0

[46] Haberer JE , Musinguzi N , Boum Y 2nd , Siedner MJ , Mocello AR , Hunt PW , et al. Duration of antiretroviral therapy adherence interruption is associated with risk of virologic rebound as determined by real‐time adherence monitoring in rural Uganda. J Acquir Immune Defic Syndr. 2015;70(4):386–92. 26110445 10.1097/QAI.0000000000000737 PMC4624495

[47] UNAIDS . Start free, stay free, AIDS free: 2017 Progress Reprot. 2018.

[48] Ware NC , Wyatt MA , Pisarski EE , Bwana BM , Orrell C , Asiimwe S , et al. Influences on Adherence to Antiretroviral Therapy (ART) in early‐stage HIV disease: qualitative study from Uganda and South Africa. AIDS Behav. 2020 [Epub ahead of print] 10.1007/s10461-020-02819-z PMC11091710 32140877

[49] Chinkonde JR , Sundby J , Martinson F . The prevention of mother‐to‐child HIV transmission programme in Lilongwe, Malawi: why do so many women drop out. Reprod Health Matters. 2009;17(33):143–51. 19523591 10.1016/S0968-8080(09)33440-0

[50] Kalichman SC , Simbayi LC , Jooste S , Toefy Y , Cain D , Cherry C , et al. Development of a brief scale to measure AIDS‐related stigma in South Africa. AIDS Behav. 2005;9(2):135–43. 15933833 10.1007/s10461-005-3895-x

[51] Tsai AC , Bangsberg DR , Weiser SD . Harnessing poverty alleviation to reduce the stigma of HIV in Sub‐Saharan Africa. PLoS Med. 2013;10:e1001557. 24319400 10.1371/journal.pmed.1001557 PMC3841100

[52] Wolfe WR , Weiser SD , Leiter K , Steward WT , Percy‐de Korte F , Phaladze N , et al. The impact of universal access to antiretroviral therapy on HIV stigma in Botswana. Am J Public Health. 2008;98(10):1865–71. 18703447 10.2105/AJPH.2007.122044 PMC2636454

[53] Katz IT , Ryu AE , Onuegbu AG , Psaros C , Weiser SD , Bangsberg DR , et al. Impact of HIV‐related stigma on treatment adherence: systematic review and meta‐synthesis. J Int AIDS Soc. 2013;16:18640. 24242258 10.7448/IAS.16.3.18640 PMC3833107

[54] Redinger S , Norris SA , Pearson RM , Richter L , Rochat T . First trimester antenatal depression and anxiety: prevalence and associated factors in an urban population in Soweto, South Africa. J Dev Orig Health Dis. 2018;9(1):30–40. 28877770 10.1017/S204017441700071X

[55] Knettel BA , Cichowitz C , Ngocho JS , Knippler ET , Chumba LN , Mmbaga BT , et al. Retention in HIV care during pregnancy and the postpartum period in the option B+ era: systematic review and meta‐analysis of studies in Africa. J Acquir Immune Defic Syndr. 2018;77(5):427–38. 29287029 10.1097/QAI.0000000000001616 PMC5844830

[56] Psaros C , Stanton AM , Bedoya CA , Mosery N , Evans S , Matthews LT , et al. Protocol for a prospective evaluation of postpartum engagement in HIV care among women living with HIV in South Africa. BMJ Open. 2020;10:e035465. 10.1136/bmjopen-2019-035465 PMC6955573 31924641

[57] Soomro N , Fitzgerald G , Seeley J , Schatz E , Nachega JB , Negin J . Comparison of antiretroviral therapy adherence among HIV‐infected older adults with younger adults in africa: systematic review and meta‐analysis. AIDS Behav. 2019;23(2):445–58. 29971732 10.1007/s10461-018-2196-0 PMC6373524

[58] Ghidei L , Simone MJ , Salow MJ , Zimmerman KM , Paquin AM , Skarf LM , et al. Aging, antiretrovirals, and adherence: a meta analysis of adherence among older HIV‐infected individuals. Drugs Aging. 2013;30(10):809–19. 23959913 10.1007/s40266-013-0107-7 PMC3844933

[59] Baeten JM , Palanee‐Phillips T , Brown ER , Schwartz K , Soto‐Torres LE , Govender V , et al. Use of a vaginal ring containing dapivirine for HIV‐1 prevention in women. N Engl J Med. 2016;375(22):2121–32. 26900902 10.1056/NEJMoa1506110 PMC4993693

[60] Steinberg L . A social neuroscience perspective on adolescent risk‐taking. Dev Rev. 2008;28(1):78–106. 18509515 10.1016/j.dr.2007.08.002 PMC2396566

[61] Adakun SA , Siedner MJ , Muzoora C , Haberer JE , Tsai AC , Hunt PW , et al. Higher baseline CD4 cell count predicts treatment interruptions and persistent viremia in patients initiating ARVs in rural Uganda. J Acquir Immune Defic Syndr. 2013;62(3):317–21. 23242160 10.1097/QAI.0b013e3182800daf PMC3696032

[62] Bock P , James A , Nikuze A , Peton N , Sabapathy K , Mills E , et al. Baseline CD4 count and adherence to antiretroviral therapy: a systematic review and meta‐analysis. J Acquir Immune Defic Syndr. 2016;73(5):514–21. 27851712 10.1097/QAI.0000000000001092

[63] Odeny TA , Onono M , Owuor K , Helova A , Wanga I , Bukusi EA , et al. Maximizing adherence and retention for women living with HIV and their infants in Kenya (MOTIVATE! study): study protocol for a randomized controlled trial. Trials. 2018;19(1):77. 29378622 10.1186/s13063-018-2464-3 PMC5789594

[64] Larson BA , Bii M , Tsikhutsu I , Halim N , Wolfman V , Coakley P , et al. The Enhanced Mentor Mother ProgrAm (EMMA) for the prevention of mother‐to‐child transmission of HIV in Kenya: study protocol for a cluster randomized controlled trial. Trials. 2018;19(1):594. 30376872 10.1186/s13063-018-2975-y PMC6208066

[65] Aliyu MH , Sam‐Agudu NA , Shenoi S , Goga AE , Ramraj T , Vermund SH , et al. Increasing male engagement in the prevention of vertical transmission of HIV: what works in sub‐Saharan Africa? BMJ. 2019;365:l1965. 31171558 10.1136/bmj.l1965 PMC6598674

[66] Aliyu MH , Blevins M , Audet CM , Kalish M , Gebi UI , Onwujekwe O , et al. Integrated prevention of mother‐to‐child HIV transmission services, antiretroviral therapy initiation, and maternal and infant retention in care in rural north‐central Nigeria: a cluster‐randomised controlled trial. Lancet HIV. 2016;3(5):e202–11. 10.1016/S2352-3018(16)00018-7 PMC4852280 27126487

[67] Myer L , Phillips TK , Zerbe A , Brittain K , Lesosky M , Hsiao NY , et al. Integration of postpartum healthcare services for HIV‐infected women and their infants in South Africa: A randomised controlled trial. PLoS Med. 2018;15:e1002547. 29601570 10.1371/journal.pmed.1002547 PMC5877834

[68] Geldsetzer P , Yapa HM , Vaikath M , Ogbuoji O , Fox MP , Essajee SM , et al. A systematic review of interventions to improve postpartum retention of women in PMTCT and ART care. J Int AIDS Soc. 2016;19:20679. 27118443 10.7448/IAS.19.1.20679 PMC4846797

[69] Odeny TA , Bukusi EA , Cohen CR , Yuhas K , Camlin CS , McClelland RS . Texting improves testing: a randomized trial of two‐way SMS to increase postpartum prevention of mother‐to‐child transmission retention and infant HIV testing. AIDS. 2014;28(15):2307–12. 25313586 10.1097/QAD.0000000000000409 PMC4834137

[70] Odeny TA , Hughes JP , Bukusi EA , Akama E , Geng EH , Holmes KK , et al. Text messaging for maternal and infant retention in prevention of mother‐to‐child HIV transmission services: a pragmatic stepped‐wedge cluster‐randomized trial in Kenya. PLoS Med. 2019;16:e1002924. 31577792 10.1371/journal.pmed.1002924 PMC6774469

[71] Schwartz SR , Clouse K , Yende N , Van Rie A , Bassett J , Ratshefola M , et al. Acceptability and feasibility of a mobile phone‐based case management intervention to retain mothers and infants from an option B+ program in postpartum HIV Care. Matern Child Health J. 2015;19(9):2029–37. 25656728 10.1007/s10995-015-1715-0 PMC4871127

[72] Yotebieng M , Thirumurthy H , Moracco KE , Edmonds A , Tabala M , Kawende B , et al. Conditional cash transfers to increase retention in PMTCT care, antiretroviral adherence, and postpartum virological suppression: a randomized controlled trial. J Acquir Immune Defic Syndr. 2016;72 Suppl 2:S124–9. 27355499 10.1097/QAI.0000000000001062 PMC5113245

[73] Drake AL , Unger JA , Ronen K , Matemo D , Perrier T , DeRenzi B , et al. Evaluation of mHealth strategies to optimize adherence and efficacy of Option B+ prevention of mother‐to‐child HIV transmission: Rationale, design and methods of a 3‐armed randomized controlled trial. Contemp Clin Trials. 2017;57:44–50. 28315480 10.1016/j.cct.2017.03.007 PMC5522580

[74] Awiti PO , Grotta A , van der Kop M , Dusabe J , Thorson A , Mwangi J , et al. The effect of an interactive weekly mobile phone messaging on retention in prevention of mother to child transmission (PMTCT) of HIV program: study protocol for a randomized controlled trial (WELTEL PMTCT). BMC Med Inform Decis Mak. 2016;16:86. 27401475 10.1186/s12911-016-0321-4 PMC4940723

[75] van Heyningen T , Honikman S , Tomlinson M , Field S , Myer L . Comparison of mental health screening tools for detecting antenatal depression and anxiety disorders in South African women. PLoS One. 2018;13:e0193697. 29668725 10.1371/journal.pone.0193697 PMC5906008

[76] Honikman S , van Heyningen T , Field S , Baron E , Tomlinson M . Stepped care for maternal mental health: a case study of the perinatal mental health project in South Africa. PLoS Med. 2012;9:e1001222. 22666181 10.1371/journal.pmed.1001222 PMC3362637

[77] Trafford Z , Gomba Y , Colvin CJ , Iyun VO , Phillips TK , Brittain K , et al. Experiences of HIV‐positive postpartum women and health workers involved with community‐based antiretroviral therapy adherence clubs in Cape Town, South Africa. BMC Public Health. 2018;18(1):935. 30064405 10.1186/s12889-018-5836-4 PMC6069812

[78] Buregyeya E , Naigino R , Mukose A , Makumbi F , Esiru G , Arinaitwe J , et al. Facilitators and barriers to uptake and adherence to lifelong antiretroviral therapy among HIV infected pregnant women in Uganda: a qualitative study. BMC Pregnancy Childbirth. 2017;17(1):94. 28320347 10.1186/s12884-017-1276-x PMC5360052

[79] Fatti G , Shaikh N , Eley B , Grimwood A . Effectiveness of community‐based support for pregnant women living with HIV: a cohort study in South Africa. AIDS Care. 2016;28 Suppl 1:114–8. 26922939 10.1080/09540121.2016.1148112 PMC4828595

[80] Collins LM , Murphy SA , Strecher V . The multiphase optimization strategy (MOST) and the sequential multiple assignment randomized trial (SMART): new methods for more potent eHealth interventions. Am J Prev Med. 2007;32 5 Suppl:S112–8. 17466815 10.1016/j.amepre.2007.01.022 PMC2062525

[81] Myer L , Iyun V , Zerbe A , Phillips TK , Brittain K , Mukonda E , et al. Differentiated models of care for postpartum women on antiretroviral therapy in Cape Town, South Africa: a cohort study. J Int AIDS Soc. 2017;20:21636. 28770593 10.7448/IAS.20.5.21636 PMC5577773

[82] Musinguzi N , Muganzi CD , Boum YI , Ronald A , Marzinke MA , Hendrix CW , et al. Comparison of subjective and objective adherence measures for preexposure prophylaxis against HIV infection among serodiscordant couples in East Africa. AIDS. 2016;30(7):1121–9. 26785125 10.1097/QAD.0000000000001024

[83] Orrell C , Cohen K , Leisegang R , Bangsberg DR , Wood R , Maartens G . Comparison of six methods to estimate adherence in an ART‐naive cohort in a resource‐poor setting: which best predicts virological and resistance outcomes? AIDS Res Ther. 2017;14(1):20. 28376815 10.1186/s12981-017-0138-y PMC5379739

